# Lightweight Co_3_O_4_/CC Composites with High Microwave Absorption Performance

**DOI:** 10.3390/nano13131903

**Published:** 2023-06-21

**Authors:** Bing An, Mei Wu, Xinhuang Yang, Zengming Man, Chunyang Feng, Xiaohui Liang

**Affiliations:** 1 Hangzhou Dianzi University, Hangzhou 310018, China; 2 Xiamen University, Xiamen 361005, China; 3 National Engineering Laboratory for Textile Fiber Materials & Processing Technology, Zhejiang Sci-Tech University, Hangzhou 310018, China

**Keywords:** low frequency, Co_3_O_4_/CC, Co-MOFs/CC, thinner thickness

## Abstract

With the rapid development of electronic and communication technology for military radars, the demand for microwave-absorbing materials in the low-frequency range with thin layers is growing. In this study, flexible Co_3_O_4_/CC (carbon cloth) composites derived from Co-MOFs (metal–organic frameworks) and CC are prepared using hydrothermal and thermal treatment processes. The flexible precursors of the Co-MOFs/CC samples are calcined with different calcination temperatures, for which the material structure, dielectric properties, and microwave absorption performance are changed. With the increases in calcination temperature, the minimum reflection loss of the corresponding Co_3_O_4_/CC composites gradually moves to the lower frequency with a thinner thickness. In addition, the Co_3_O_4_/CC composites with the 25 wt% filler loading ratio exhibit the minimum reflection loss (RL) of −46.59 dB at 6.24 GHz with a 4.2 mm thickness. When the thickness is 3.70 mm, the effective absorption bandwidth is 3.04 GHz from 5.84 to 8.88 GHz. This study not only proves that the Co_3_O_4_/CC composite is an outstanding microwave-absorbing material with better flexibility but also provides useful inspiration for research on wideband microwave absorption materials below 10 GHz.

## 1. Introduction

With the rapid development of science and technology, the influences of electromagnetic radiation on the human body and environment have attracted extensive attention. In terms of this background, the development of microwave absorption materials can help resist electromagnetic radiation and has become a research hotspot around the world [1]. An ideal microwave-absorbing material should have excellent properties such as a light weight, thin thickness, strong reflection, and broad effective absorption bandwidth [2]. In recent years, researchers have studied a variety of ideal microwave-absorbing materials including magnetic loss materials and dielectric loss materials. For instance, Wang et al. synthesized Fe_3_O_4_@SnO_2_/RGO ternary composites with an efficient and rapid three-step method, improving the impedance matching and obtaining excellent electromagnetic wave absorption performance [3]. Moreover, ZnO [4], Fe_2_O_3_/N-Graphene/CNTs [5], composite nonlinear feedback (CNF)/SiO_2_ [6], and MOF derivatives [7] have also been widely developed to improve the attenuation characteristics of electromagnetic waves. In view of the loss mechanism of electromagnetic waves, it is generally easier to achieve strong broadband microwave absorption at high frequencies. However, the absorbing materials at low frequencies are normally thick and have a narrow frequency bandwidth, making it difficult to meet the needs of practical applications. Therefore, it is essential to research lightweight, thinner layers with wide bandwidth absorbers below 10 GHz.

Among a large number of lightweight electromagnetic absorption materials, MOF derivatives have been extensively manufactured because of their variety of metals, diverse morphologies, and large surface areas [8,9,10,11]. For example, Ma et al. synthesized TiO_2_/C nanoporous carbon composites via the pyrolysis of MIL-125 (Ti-based MOFs), which exhibited a maximum reflection loss of −49.60 dB, with a thinner thickness of 1.6 mm and obtaining a 4.6 GHz effective absorption bandwidth [12]. In addition, Shi et al. studied the nanoporous Co_3_O_4_ nanosheets derived from Co-MOFs. When the matching thickness is 2.5 mm, the maximum reflection loss can reach −32 dB, and the effective absorption band width can reach 4.2 GHz because of its better impedance matching of the composites [13]. Additionally, Liu et al. also designed Co-MOF derivatives to improve the absorption performance at low frequencies. Better impedance matching was achieved with the Co/C composite, the minimum reflection loss value for which was about −20 dB, while the effective absorption bandwidth reached 3.84 GHz in the C-band frequency range [14]. Therefore, the MOF derivatives could obtain broadband absorption performances with thinner thicknesses at low frequencies. In addition, MOFs can be combined with other functional materials to improve the microwave absorption performance. For example, Yang et al. fabricated SiC/Ni/NiO/C by annealing SiC/Ni-MOFs nanoparticles, for which the minimal RL value reached −50.52 dB [15]. Furthermore, Fe/Fe_3_C/C derived from MIL-101-Fe and MIL-88-Fe was obtained by Miao et al. The minimum RL of the composites was −59.2 dB with a thickness of 4.32 mm, and the effective bandwidth reached 6.5 GHz at 2 mm [16]. Thus, the combination of MOF derivatives and another material is better for the loss of microwave absorption.

In recent decades, carbon cloth (CC) has been applied to various fields owing to its light weight, high strength, corrosion resistance, and aging resistance [17,18,19,20,21,22,23]. Luo et al. prepared NiSe_2_/CC as an electrode material without an adhesive, which showed good hydrogen evolution reaction performance and chemical stability in a 0.5 mol/LH_2_SO_4_ solution [24]. Zhan et al. grew porous arrays of CoP nanoparticles derived from MOFs on carbon cloth for effective alkaline hydrogen evolution [25]. Along with these studies, carbon cloth can also be used to prepare flexible microwave absorbers. Che et al. made ZnO grow on a CC flexible substrate, constructing a three-dimensional conductive network with multiple interfaces [26]. Moreover, Liu et al. grew Co-MOFs on a conductive CC and derived CC@NPC/CoS_2_ composites of RL reaching −59.6 dB at 2.8 mm [27]. Based on the above, the carbon cloth can be combined with metal–organic framework materials to prepare excellent electromagnetic wave absorbers with thin layers.

In this study, Co_3_O_4_/CC derived from Co-BTC-MOFs/CC (Co-MOFs/CC) was synthesized, with the results indicating that the absorbing properties of the Co_3_O_4_/CC composite can be adjusted by controlling the different calcination temperatures. When the calcination temperature was 500 °C, the Co_3_O_4_/CC composites showed an RL_min_ (minimum reflection loss) of −46.59 dB at 6.24 GHz, with a filler loading ratio of 25 wt%. Furthermore, the Co_3_O_4_/CC material exhibited an effective microwave absorption bandwidth of 3.04 GHz from 5.84 GHz to 8.88 GHz with a thickness of 3.70 mm. Thus, the Co_3_O_4_/CC composite derived from the metal–organic framework and CC can be seen as a promising wide-band absorbing material below 10 GHz with a thin layer.

## 2. Materials and Methods

### 2.1. Preparation of the Co_3_O_4_/CC Precursor

**Figure 1:**
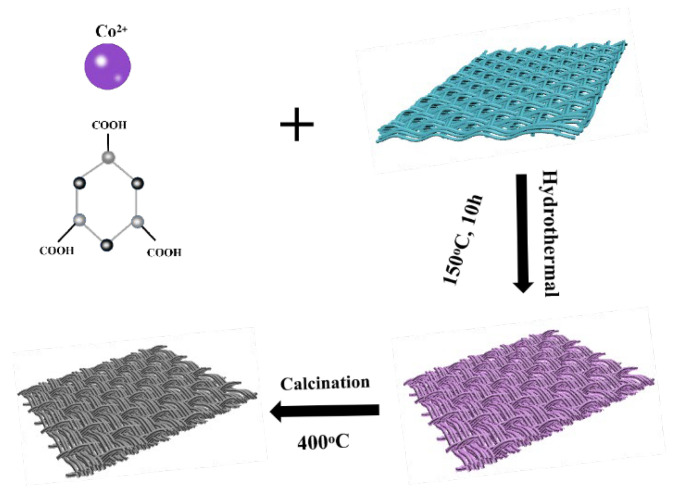
A structure diagram of the experimental process.

A piece of 2 × 2 cm^2^ CC was placed in acetone for cleaning by deionized water and ethanol, which was ultrasonicated for 20 min and then dried in a vacuum oven at 60 °C for 6 h. The preparation of the Co-MOFs/CC occurred as in Figure 1, by dissolving 432 mg of cobalt nitrate hexahydrate, 300 mg of trimesic acid, and 3 g of PVP K30 (polyvinylpyrrolidone K30) in a 60 mL mixture solution (deionized water/DMF/ethanol =1:1:1 *v*/*v*/*v*) and stirring vigorously with a blender until completely dissolved. Then, we placed the cleaning CC into the bottom of a 100 mL Teflon autoclave and transferred the solution into it. Finally, the Teflon autoclave was heated in an oven at 150 °C for 10 h and the Co-MOFs/CC was obtained. The Co_3_O_4_/CC precursor was obtained after washing with deionized water and ethanol three times and drying in an oven at 60 °C for 12 h.

### 2.2. Synthesis of Co_3_O_4_/CC Composites

The Co-MOFs/CC composites were heated to 300 °C, 400 °C, and 500 °C, respectively, in a muffle furnace with a heating rate of 2 °C/min and heated for two hours to obtain multilayer Co_3_O_4_/CC composites, named as S1, S2, and S3, respectively.

### 2.3. Characterization and Measurement

The morphologies of the Co_3_O_4_/CC and pure CC were characterized by field emission scanning electron microscopy (FE-SEM, S-4800, Hitachi, Tokyo, Japan), and an X-ray diffractometer (XRD) was used to collect powder diffraction data in the range of 10–70 °C with Cu Kα radiation (λ = 1.5418 Å). The XPS data were obtained using a model Versa Probe (PHI 5000, Guangzhou, China). Then, the relative complex permittivity and permeability in the frequency range of 2–18 GHz were measured by the vector network analyzer (Agilent PNA N5224A, Keysight, Budd Lake, NJ, USA) with the coaxial method. The filler loading ratio of the testing ring was 25 wt%, and the inner and external diameters were 3.04 and 7.00 mm, respectively. The microwave absorption performance with reflection loss of the Co_3_O_4_/CC was calculated by the permittivity and permeability.

## 3. Results and Discussion

**Figure 2:**
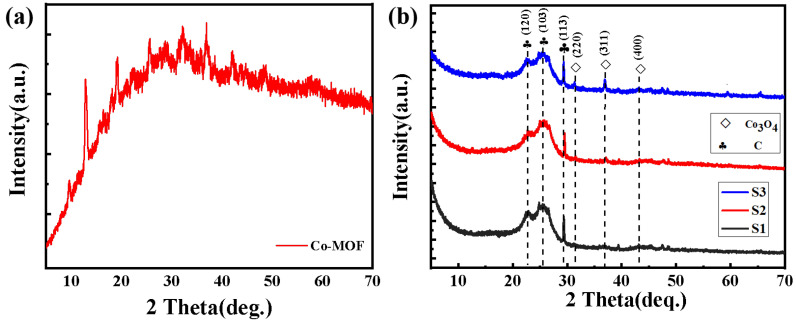
XRD pattern of Co-MOFs (**a**) and the XRD patterns of S1–S3 (**b**).

The sample phase composition and crystallinity of Co_3_O_4_/CC composites can be observed using an XRD diffraction analysis. In order to test the successful synthesis of the Co-MOFs and Co_3_O_4_/CC composites, we firstly studied the XRD patterns of the samples before and after calcination. Figure 2a is the XRD pattern of the Co-MOFs/CC, and obvious diffraction peaks can be observed at 12.73°, 19.21°, 28.70°, 32.32°, and 36.95°, which fully coincide with the locations of the Co-MOFs’ diffraction peaks [28,29]. This proves the successful synthesis of the precursor Co-MOFs. Figure 2b shows the XRD pattern of S1–S3 obtained from powder diffraction data processed by the X-ray diffractometer in the region of 10 °C to 70 °C. The diffraction peaks of Co_3_O_4_ can be observed at 31.27°, 36.85°, and 44.81° for S1–S3, corresponding to the (220), (311), and (400) lattice planes, respectively. In addition, obvious diffraction peaks of C can be observed at 22.76° (120), 26.57° (103), and 29.31° (113), which prove the existence of Co_3_O_4_ and carbon [30]. At the same time, no other impurity peaks can be observed, indicating that the sample has high purity and crystallinity [31]. It is obvious that Co_3_O_4_/CC was successfully synthesized.

**Figure 3:**
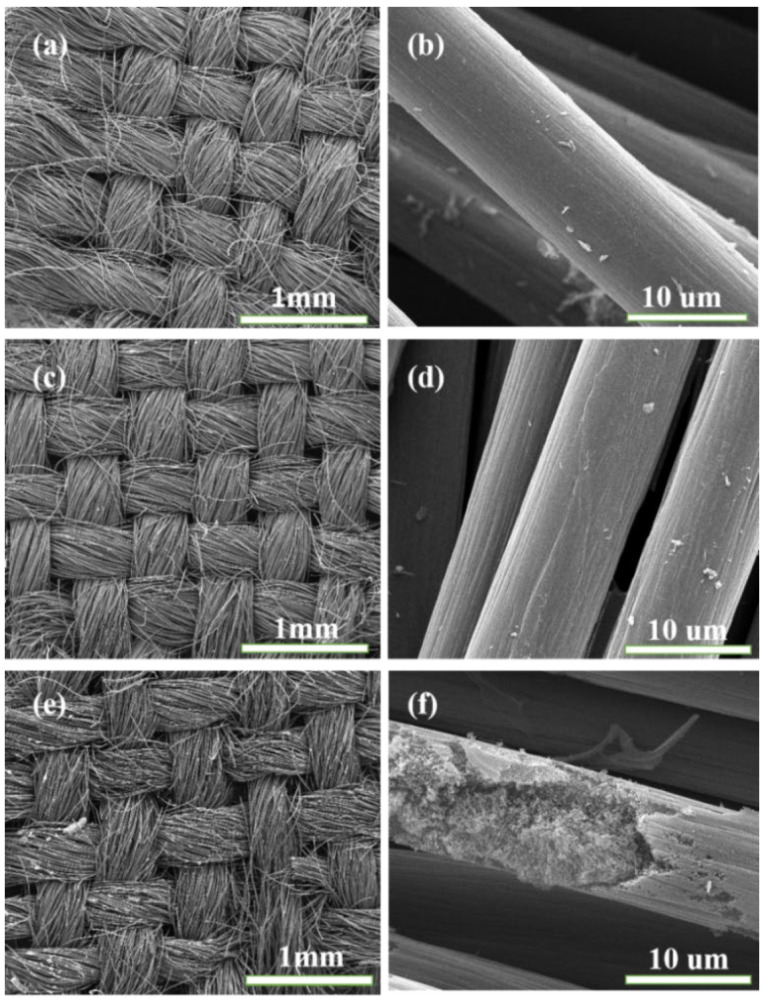
SEM images of (**a**,**b**) S1, (**c**,**d**) S2, and (**e**,**f**) S3.

The pictures of CC, Co-MOFs/CC, and Co_3_O_4_/CC are shown in Figure S1, showing that the Co_3_O_4_/CC kept the flexibility of the CC. The morphologies of Co_3_O_4_/CC composites with different temperatures were characterized by SEM pictures, which are shown in Figure 3. It can be seen from Figure 3 that the morphologies of the samples with the three different temperatures are very similar. The cross-arrangement of the cylindrical morphology is well maintained and the structure’s size is quite uniform, which is better for the reflection and scattering of electromagnetic waves, which could improve the microwave absorption performance. However, with increasing temperatures, the morphology of the Co_3_O_4_/CC becomes rough. In particularly, it can be seen from Figure 3f that most of the cylinder has been broken and presents a loose and porous structure, which may be caused by the collapse of the Co-MOFs. In addition, the surface of the CC is relatively smooth and a floccule appears in the broken CC, which demonstrates that the Co_3_O_4_ is grown inside of the CC. The floccule could provide a loose and porous structure, which can offer more contact sites and increase the attenuation of electromagnetic waves.

**Figure 4:**
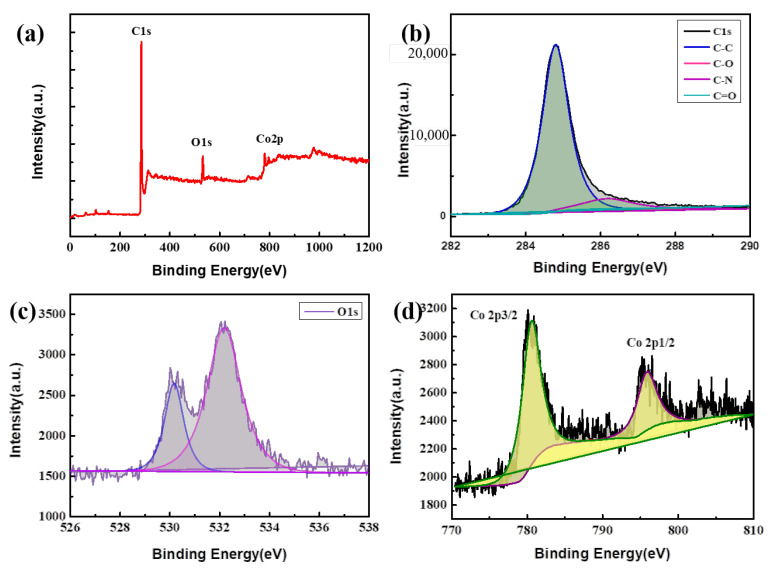
(**a**) XPS survey spectrum of S3. (**b**) C 1s, (**c**) O 1s, and (**d**) Co 2p from the core-level spectrum in S3. (Different colors represent different peak positions).

The valence states of elements in the composites can be clearly indicated by XPS [32]. The XPS results for the Co_3_O_4_/CC are shown in Figure 4. Figure 4a shows the wide spectrum of Co_3_O_4_/CC composite materials, while Figure 4b–d show the spectra of C 1s, O 1s, and Co 2p of the S3 composite, respectively. As can be seen from Figure 4b, two characteristic peaks appear in the C-1s spectrum of Co_3_O_4_/CC, which was peak-divided, with the C-C/C=C peak appearing at 284.6 eV and the C-O peak emerging at 286.7 eV. It is obvious that the C-C/C=C bond signal is the strongest, which indicates that most of the Co_3_O_4_/CC precursor has become amorphous carbon after calcination and carbonization [33]. The results of the XPS analysis in Figure 4c show that two characteristic peaks also appear in the O 1s spectrum of Co_3_O_4_/CC, in which the peaks loading at 531.6 eV and 532.5 eV are the chemisorbed oxygen and the lattice O of Co_3_O_4_. In Figure 4d, the spectral peaks of Co 2P at 781.0 eV and 797.1 eV belong to Co 2P 3/2 and Co 2P 1/2, respectively [34]. The characteristic peaks can be attributed to cobalt nitrate hexahydrate and also indicate the presence of C and O elements.

**Figure 5:**
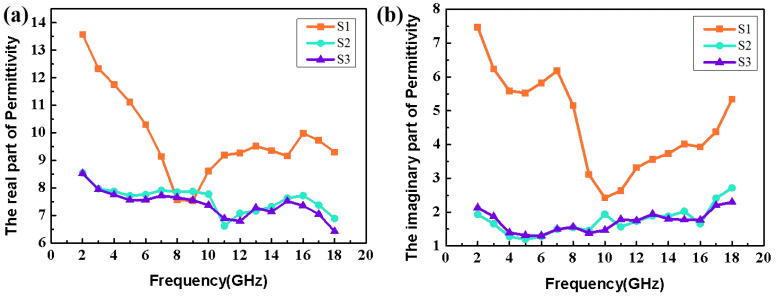
The permittivity of the real (**a**) and imaginary (**b**) parts of the Co_3_O_4_/CC complex at three temperatures.

After calcination of the Co_3_O_4_/CC precursor at different temperatures, the internal structure of the Co_3_O_4_/CC composite will be changed, which will affect the dielectric constant and magnetic permeability of the composite and adjust its microwave absorption properties. In this study, we only pay attention to the dielectric characteristic analysis on account of the weak magnetism of the generated Co_3_O_4_/CC complex. The real parts (ε′) and imaginary parts (*ε*″) of the complex dielectric constant represent the dielectric storage and dielectric loss capacity of the material, respectively [35]. It can be revealed from Figure 5 that the *ε*′ of the Co_3_O_4_/CC complex generally decreases with the increase in frequency. It is worth noting that the ε′ increases several times after the high frequency part at 9 GHz, which is mainly from exchange resonance [36]. According to Debye’s theory, the real and imaginary parts of the complex dielectric constant can be expressed in the following forms [37]:

(1) $$\varepsilon^{\prime }{=\varepsilon }_{\infty }{+(\varepsilon }_{s}-\varepsilon_{\infty }{)/(1+\omega }^{2}\tau^{2})$$

(2) $$\varepsilon^{\prime \prime }{=(\varepsilon }_{s}-\varepsilon_{\infty }{)\omega \tau /(1+\omega }^{2}\tau^{2}{)+\sigma }_{\mathrm{ac}}{/\omega \varepsilon }_{0}$$

where *ε_s_* represents the static permittivity, *ε_∞_* is the infinite static permittivity, *ω* is the angular frequency, *τ* is the polarization relaxation time, *σ_ac_* is the electrical conductivity, and *ε*_0_ represents the vacuum permittivity. According to Equation (1), the real part of the dielectric constant of the generated complex decreases due to the increase in angular frequency, which reveals the dispersion effect of the complex in favor of microwave absorption. The essence of this phenomenon is from the existence of polarization relaxation below this frequency. Moreover, the peak value of the imaginary part of the dielectric constant (*ε*″) belongs to the dielectric polarization peak, which is conducive to increasing the dielectric polarization loss. In fact, *ε*″ is an expression of the material’s electrical conductivity, based on Debye’s theory, which can be expressed by using the following formula [37]:

(3) $$\varepsilon^{\prime \prime }\approx \frac{\sigma }{2\pi \varepsilon_{0}f}$$

where σ is the electrical conductivity and $\varepsilon_{0\;}$ is the dielectric constant in a vacuum. According to Formula (3), the decrease in conductivity leads to a lower *ε*″, which indicates that the complex S3 has lower electrical conductivity than S1 and S2. This is because more and more Co_3_O_4_ is generated as the temperature increases, resulting in an increase in the resistivity of the material. From Figure 5b, it can be seen that the *ε*″ order of the complex at the three temperatures is roughly S1 > S2 > S3, indicating that except for the polarization relaxation phenomenon, there are conductivity losses due to the migration of free carriers in the CC.

**Figure 6:**
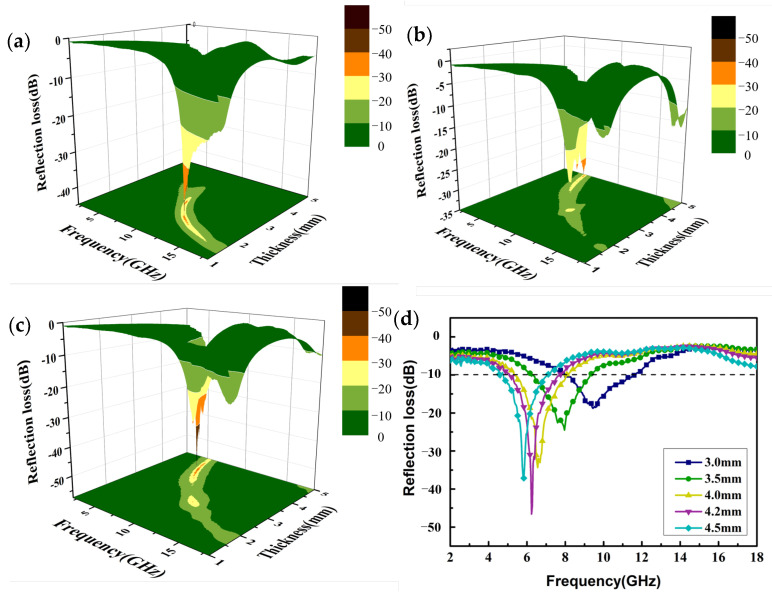
The 3D plots of RL values for Co_3_O_4_/CC samples with different calcination temperatures of (**a**) S1, (**b**) S2, and (**c**) S3 in the frequency range of 2–18 GHz. (**d**) The RL values of S3 with different thicknesses.

After analyzing the complex permittivity of the Co_3_O_4_/CC composite, the reflection loss of the generated composite was analyzed. Figure 6 shows a three-dimensional image of the RLs with different calcination temperatures and matching thicknesses, where one can directly observe the *R_L_* value of the Co_3_O_4_/CC composite. The calculation formulas for the *R_L_* of the electromagnetic wave are as follows:

(4) $$Z_{in}=Z_{0}{\left(\mu_{r}/\varepsilon_{r}\right)}^{1/2}\tan\;h\left[j\left(2\pi fd/c\right)\;{\left(\varepsilon_{r}\mu_{r}\right)}^{1/2}\right]$$

(5) $$R_{L}=20\log\left(Z_{in}-Z_{0}\right)/\left(Z_{in}+Z_{0}\right)$$

where *Z*_0_ represents the impedance value of the free space. Figure 6a shows the three-dimensional (3D) *R_L_* of S1, and it can be seen that the minimum *R_L_* is −44.68 dB at 11.72 GHz with a thickness of 2.10 mm. Although the matching thickness of S1 is relatively thin, the frequency corresponding to the minimum reflection loss is still in the high-frequency range (Ku band). The minimum reflection loss of S2 in Figure 6b is −33.95 dB, and the corresponding frequency and thickness are 5.4 GHz and 4.90 mm. The *R_L_* value of S3 is shown in Figure 6c, and the minimum *R_L_* can reach −46.59 dB at 6.24 GHz with a 4.2 mm thickness. It can be concluded that the frequency of the minimum *R_L_* gradually moves to low frequencies with the increase in calcination temperature. According to Figure 6a, the RL value is less than −10 dB in the frequency range of 10.76 to 14.16 GHz for S1; thus, the effective absorption band width of S1 is 3.4 GHz. Similarly, S2 (Figure 6b) has an effective absorption band width of 1.88 GHz in the frequency range of 4.52~6.4 GHz, while S3 (Figure 6d) has an effective absorption band width of 2.6 GHz with a frequency range of 5.12~7.72 GHz. It is well known that a good electromagnetic wave absorber should have good attenuation ability, superior impedance matching performance, a minimal RL value, a wide effective absorption band width, and so on [38,39]. According to the above analysis, it is obvious that S3 shows better reflection loss and a thinner matching thickness in the frequency region of less than 6 GHz. Therefore, the manufacture of Co_3_O_4_/CC complexes at a calcination temperature of 500 °C is suitable for microwave-absorbing materials in the low-frequency range.

In order to further explore the reason why the Co_3_O_4_/CC composites have different absorbing properties with different calcination temperatures, the dielectric loss, attenuation ability for electromagnetic waves, and impedance matching of the composites were further analyzed in this study. The dielectric loss is usually presented by the loss tangent of permittivity, with a higher loss tangent indicating better dielectric loss. The loss tangent of permittivity can be expressed in the following formula [40]:

(6) $${\tan\delta }_{\varepsilon }=\varepsilon^{\prime \prime }/\varepsilon^{\prime }$$

**Figure 7:**
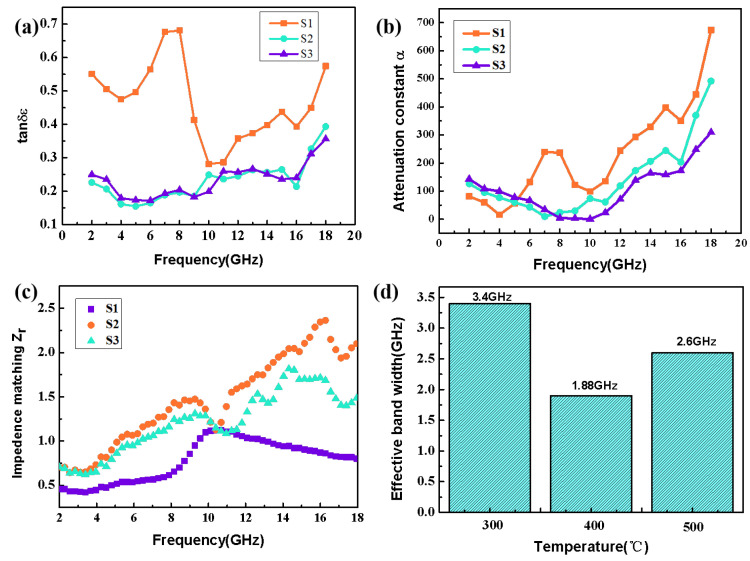
Frequency dependence rates of (**a**) tan δ_ε_, (**b**) α, and (**c**) Z_r_ for S1–S3. (**d**) Effective absorption bandwidths with three calcination temperatures.

Figure 7a presents the variations in dielectric loss of the Co_3_O_4_/CC composites with different calcination temperatures. In general, the tan *δ_ε_* values at three temperatures demonstrate an upward trend in terms of the increase in frequency. Additionally, a few relaxation peaks appear at low frequencies, which proves that polarization relaxation occurs in the low-frequency range, indicating better microwave absorption. Regarding samples S1–S3, the S1 composite has higher dielectric loss.

In addition to the dielectric loss, the attenuation factor α [41] and impedance matching *Z_r_* [42] also play vital roles in the electromagnetic wave absorption performance. Attenuation α refers to the amplitude or power attenuation in the electromagnetic wave transmission process, which is related to the dielectric loss. As can be seen from Figure 7b, the magnitude relationship of the attenuation ability of the three samples is α_S1_ > α_S2_ > α_S3_, indicating that sample S1 has better attenuation ability for electromagnetic waves. This is because with the enhanced calcination temperature, the amount of Co_3_O_4_ generated gradually increases, leading to a decrease in permittivity. This phenomenon will bring about decreased dielectric loss and attenuation loss, illustrating that the decrease in attenuation α is connected with the increase in calcination temperatures. For electromagnetic absorbents, an outstanding absorbing material should consider not only the strong electromagnetic propagation loss but also the impedance matching. The attenuation constant α and impedance matching *Z_r_* can be described in the following formulas:

(7) $$\alpha =\frac{\sqrt{2}\pi f}{c}\sqrt{(\mu^{\prime \prime }\varepsilon^{\prime \prime }-\mu^{\prime }\varepsilon^{\prime })+\sqrt{{\left(\mu^{\prime \prime }\varepsilon^{\prime \prime }-\mu^{\prime }\varepsilon^{\prime }\right)}^{2}+{\left(\mu^{\prime }\varepsilon^{\prime \prime }+\mu^{\prime \prime }\varepsilon^{\prime }\right)}^{2}}}$$

(8) $$Z_{r}{=Z}_{\mathrm{in}}{/Z}_{0}$$

where *Z_r_* represents the impedance matching value, *Z*_0_ is the free-space impedance value, *Z_in_* represents the incident impedance matching value, and c represents the speed of light. When *Z_r_* = 1, the electromagnetic wave can achieve zero reflection on the absorber surface; that is, the absorption effect reaches the best value and the condition of *ε_r_* = *μ_r_* should be satisfied. According to Figure 7c, the frequency corresponding to the minimum reflection loss of S1 is 11.72 GHz, and the corresponding impedance match at this frequency is 1.06. Similarly, the impedance values matching corresponding values of S2 and S3 are 0.53 and 1.00 with the minimum reflection loss, respectively. In addition, the impedance matching values of S3 are closer to 1 at 6–8 GHz. Therefore, this illustrates that S3 achieved almost perfect impedance matching at low frequencies. On the basis of this result, S3 shows better electromagnetic wave absorption performance and attenuation ability at low frequencies. Additionally, as shown in Figure 7d, S1 has the better effective broadband absorption bandwidth of the composites with the three different calcination temperatures.

**Figure 8:**
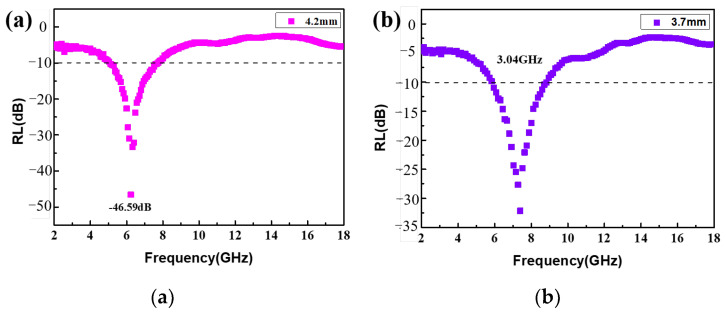
RL curves of S3 at thicknesses of (**a**) 4.2 mm and (**b**) 3.7 mm.

Therefore, S3 shows excellent microwave absorption performance below 10 GHz, and the minimum reflection loss and effective band absorption width of S3 are further analyzed in this paper. Figure 8a,b show the minimum RL values of S3 with different matching thicknesses. When the thickness is 4.2 mm, the minimum reflection loss at 6.24 GHz is −46.59 dB, indicating elegant electromagnetic wave absorption characteristics. When the thickness of S3 is reduced to 3.7 mm, the maximum effective absorption bandwidth is 3.04 GHz from 5.84 GHz to 8.88 GHz, and at that moment the minimum reflection loss is −32.13 dB. Compared with the previous work that is reported in Table 1, the Co_3_O_4_/CC composites have a thinner matching thickness and stronger reflection loss below 10 GHz.

**Figure 9:**
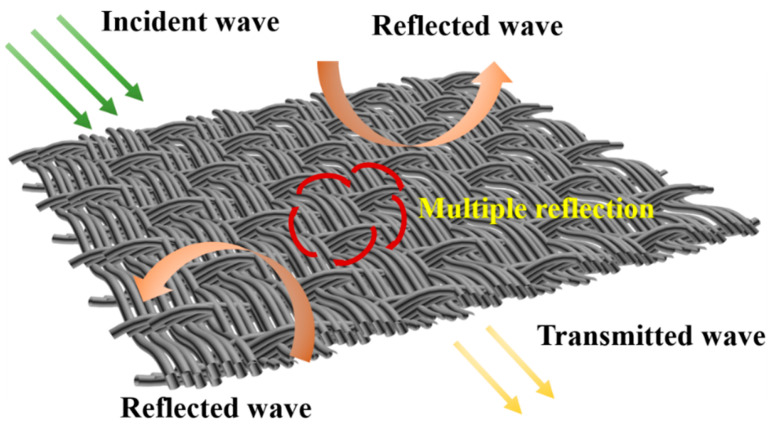
Diagram of the microwave attenuation mechanism for Co_3_O_4_/CC composites.

Based on the above, the Co_3_O_4_/CC composites have better microwave absorption properties with thinner thicknesses and have widely effective absorption bandwidths below 10 GHz. The possible mechanisms of the microwave absorption are as shown in Figure 9. Firstly, the carbon cloth and Co_3_O_4_ together cause losses of incident electromagnetic waves. Secondly, the carbon cloth could provide the network structure that allows more incident waves to enter into the internal section. Finally, when the incident wave passes into the network structure, the Co_3_O_4_ and CC could consume it and generate dielectric loss. Therefore, the CC and Co_3_O_4_ synergistically improve the microwave absorption.

## 4. Conclusions

In summary, the Co_3_O_4_/CC precursor was synthesized using the simple hydrothermal method and the Co_3_O_4_/CC derivatives were generated at different calcination temperatures. It was concluded that with the increases in calcination temperature, the electromagnetic wave absorption performance was improved gradually in the low-frequency range. When the calcination temperature was 500 °C, the minimum reflection loss RL reached −46.59 dB at 6.24 GHz with a 4.2 mm thickness. When the thickness was 3.7 mm, the effective absorption band width was 3.04 GHz in the C band and the minimum reflection loss was −32.13 dB. The synergistic effect of CC and Co_3_O_4_ resulted in better microwave absorption performance with a thinner thickness and widely effective bandwidth below 10 GHz. This provides useful inspiration for the growth of absorbing materials on carbon cloth at low frequencies, which is worthy of further investigation.

## Figures and Tables

**Table 1:** Microwave absorption performances of the similar materials.

*Filler*	*R_L_* (dB)	*Thickness* (mm)	*Effective Bandwidth* (GHz)	*Ref.*
(Zn_0.8_Mn_0.2_)_2_Y and Co_2_Z department hexagonal ferrite	−15	>5	3.2	[40]
NiZn Spinel type ferrite	−10	12	0.7	[41]
Magnesium doped barium ferrite	−10	6.0	3	[13]
Mg doped lithium zinc ferrite	−39	5.5	6.5	[13]
Co_3_O_4_/CC	−46.59	4.2	3.04	**This work**

## Data Availability

The data that support the findings of this study are available from the corresponding authors upon reasonable request.

## Supplementary Materials

The following supporting information can be downloaded at: https://www.mdpi.com/article/10.3390/nano13131903/s1, Figure S1: Photos of pure carbon cloth (a); photograph of dried CC covered with precursors (b); Photograph of CC covered with compound Co3O4/CC after calcination (c).
