# Supplementary material for: Lightweight Co3O4/CC Composites with High Microwave Absorption Performance
Source: Nanomaterials (Basel). 2023 Jun 21;13(13):1903. doi: 10.3390/nano13131903 (PMC10343397; doi:10.3390/nano13131903)
Supplement: Supplementary file 1 [file nanomaterials-13-01903-s001.zip › nanomaterials-2451723-supplementary.pdf]

Supporting Information

Article

Lightweight Co_3O_4/CC Composites with High Microwave Absorption Performance

Bing An [1,2], Mei Wu [1], Xinhuang Yang [1], Zengming Man [3], Chunyang Feng [1,*] and Xiaohui Liang [1,*]

[1] Hangzhou Dianzi University, Hangzhou 310018, China
[2] Xiamen University, Xiamen 361005, China
[3] National Engineering Laboratory for Textile Fiber Materials & Processing Technology, Zhejiang Sci-Tech University, Hangzhou 310018, China
* Correspondence: sam@hdu.edu.cn (C.F.); xhliang@hdu.edu.cn (X.L.)

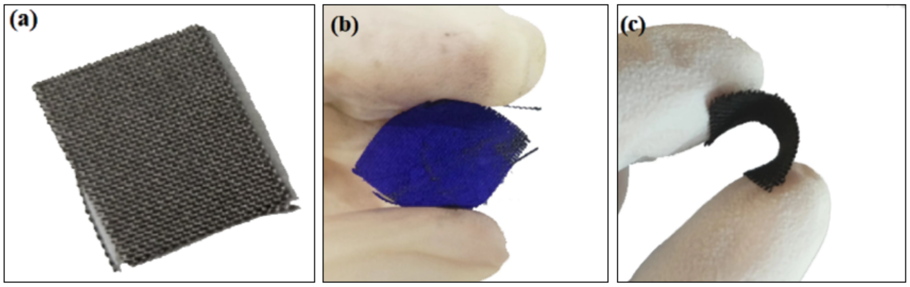

Figure S1. Photos of pure carbon cloth (**a**); photograph of dried CC covered with precursors (**b**); Photograph of CC covered with compound Co_3O_4/CC after calcination (**c**).
